# Identification of a staphylococcal complement inhibitor with broad host specificity in equid *Staphylococcus aureus* strains

**DOI:** 10.1074/jbc.RA117.000599

**Published:** 2018-02-05

**Authors:** Nienke W. M. de Jong, Manouk Vrieling, Brandon L. Garcia, Gerrit Koop, Matt Brettmann, Piet C. Aerts, Maartje Ruyken, Jos A. G. van Strijp, Mark Holmes, Ewan M. Harrison, Brian V. Geisbrecht, Suzan H. M. Rooijakkers

**Affiliations:** From the ‡ Department of Medical Microbiology, University Medical Center Utrecht, Utrecht University, 3584 CX Utrecht, The Netherlands,; the § Roslin Institute, University of Edinburgh, Easter Bush Campus, Midlothian EH25 9RG, United Kingdom,; the ¶ Department of Biochemistry and Molecular Biophysics, Kansas State University, Manhattan, Kansas 66506,; the ‖ Department of Farm Animal Health, Faculty of Veterinary Medicine, Utrecht University, 3584 CL Utrecht, The Netherlands,; the ** Department of Veterinary Medicine, University of Cambridge, Cambridge CB3 0ES, United Kingdom, and; the ‡‡ Department of Medicine, University of Cambridge, Addenbrooke's Hospital, Cambridge CB2 0QQ, United Kingdom

**Keywords:** complement system, innate immunity, Staphylococcus aureus (S. aureus), host-pathogen interaction, microbial pathogenesis, equine, host adaptation, immune evasion, SCIN

## Abstract

*Staphylococcus aureus* is a versatile pathogen capable of causing a broad range of diseases in many different hosts. *S. aureus* can adapt to its host through modification of its genome (*e.g.* by acquisition and exchange of mobile genetic elements that encode host-specific virulence factors). Recently, the prophage φSaeq1 was discovered in *S. aureus* strains from six different clonal lineages almost exclusively isolated from equids. Within this phage, we discovered a novel variant of staphylococcal complement inhibitor (SCIN), a secreted protein that interferes with activation of the human complement system, an important line of host defense. We here show that this equine variant of SCIN, eqSCIN, is a potent blocker of equine complement system activation and subsequent phagocytosis of bacteria by phagocytes. Mechanistic studies indicate that eqSCIN blocks equine complement activation by specific inhibition of the C3 convertase enzyme (C3bBb). Whereas SCIN-A from human *S. aureus* isolates exclusively inhibits human complement, eqSCIN represents the first animal-adapted SCIN variant that functions in a broader range of hosts (horses, humans, and pigs). Binding analyses suggest that the human-specific activity of SCIN-A is related to amino acid differences on both sides of the SCIN-C3b interface. These data suggest that modification of this phage-encoded complement inhibitor plays a role in the host adaptation of *S. aureus* and are important to understand how this pathogen transfers between different hosts.

## Introduction

The Gram-positive bacterium *Staphylococcus aureus* has become a major risk for both human and animal health. Whereas the bacterium harmlessly colonizes 30% of healthy adults, it can also cause severe infections ranging from abscesses to endocarditis, sepsis, and toxic shock syndrome (1). Furthermore, *S. aureus* also colonizes and infects a broad range of animal species, including cows, sheep, goats, poultry, rabbits, and horses (2). For example, *S. aureus* is a major cause of intramammary infections in dairy cows and therefore a major economic burden for the dairy industry (3). In horses, *S. aureus* can cause both community-onset infections (joint, skin, or soft tissue infections (4)) and severe surgical site infections in hospitalized horses.

Previous studies on human *S. aureus* isolates have shown that this pathogen is highly versatile and can successfully adapt to its host and various host tissues via production of specific virulence factors. For instance, *S. aureus* expresses both surface-attached and secreted proteins that allow the bacterium to adhere to various human tissues, directly kill host cells, or block important immune mechanisms (5). Some of these factors are encoded by mobile genetic elements, such as bacteriophages and pathogenicity islands, which allow more rapid acquisition and exchange of virulence genes between strains. In the past, our group was involved in the identification of a *S. aureus* bacteriophage that encodes four human-specific immune evasion molecules: the staphylococcal complement inhibitor (SCIN,^4^ referred to as SCIN-A hereafter), chemotaxis inhibitory proteins of staphylococci, staphylokinase, and staphylococcal enterotoxin A. This prophage φSa3, which specifically inserts in the β-hemolysin gene, was only found in human *S. aureus* isolates (prevalence of 90% from a genetically diverse clinical *S. aureus* strain collection (6)) and encodes secreted proteins that solely block human immune functions. The fact that this phage (and its immune evasion cluster) is lost in animal-associated strains (7, 8) suggests that this cluster is important for *S. aureus* infection in humans (6). The gene with the highest prevalence on φSa3 is *scn*, which encodes SCIN, a 9.8-kDa α-helical molecule that specifically blocks activation of the human complement system (9).

Complement is a protein network in plasma that labels bacterial cells for phagocytosis by human immune cells. This labeling is achieved via specific activation of the complement reaction on the bacterial cell (via pattern recognition molecules) that subsequently drives formation of surface-bound protease complexes (C3 convertases) that cleave C3, the major complement protein in blood. Conversion of C3 into the reactive C3b fragment results in covalent surface deposition of C3b molecules that are recognized by complement receptors on phagocytic cells (10). The SCIN-A molecule, which is secreted by *S. aureus*, specifically binds and blocks C3 convertases via a unique inhibitory mechanism that is well-characterized via structural studies (11–13). Functional studies showed that SCIN-A is highly specific for human complement, because it does not block complement activation in sera of other animals (mouse, rat, dog, sheep, guinea pig, goat, and cow) (9).

Here we identify a novel variant of SCIN that is present in *S. aureus* isolates from horses and encoded by an equid-specific prophage (termed φSaeq1). This equine SCIN molecule (eqSCIN) represents the first animal-adapted SCIN variant, because it inhibits C3 convertases of horses and other animals. These data suggest that *S. aureus* can adapt to other hosts by modification of phage-encoded complement evasion proteins.

## Results

### eqSCIN is encoded on prophage φSaeq1

**Figure 1.**
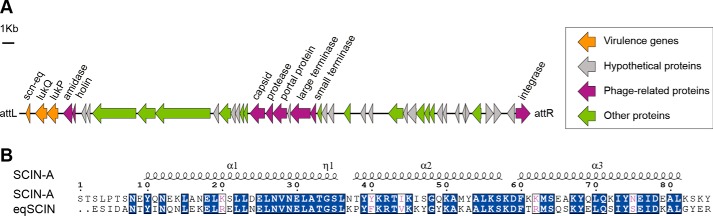
**eqSCIN is a novel SCIN variant encoded by prophage φSaeq1.** *A*, location of the *scn-eq* gene on prophage φSaeq1 of equine CC133 reference isolate 3711. *B*, amino acid alignment of eqSCIN (strain 3711) with SCIN-A (Newman) shows that these SCIN variants share 57.8% sequence identity. Conserved regions are *highlighted*.

Recently, a 45-kb prophage φSaeq1 was discovered in *S. aureus* clonal complex (CC)133 isolates from horses and donkeys that encodes the equine-specific leukocidin LukPQ (14), a bi-component pore-forming toxin consisting of LukP and LukQ subunits (Fig. 1*A*). Interestingly, LukPQ has an enhanced ability to kill equine host cells as compared with related staphylococcal leukocidins located elsewhere in the genome. Sequence analysis of CC133 reference isolate 3711 revealed that φSaeq1 also encodes a novel variant of SCIN-A (termed eqSCIN) that shares 57.8% amino acid identity with φSa3-encoded SCIN-A (Fig. 1*B*). The gene for eqSCIN (*scn-eq*) is located downstream of *lukQ* on the outer end of the prophage near the attL phage attachment site (Fig. 1*A*). Previously, we found *lukPQ* and φSaeq1 to be associated with equid strains of *S. aureus*. Here, we set out to investigate the distribution of *scn-eq*. BLASTn analysis of our collection of sequenced isolates (14) revealed that *scn-eq* was present in 29 strains from five different clonal complexes (CC1, CC133, CC522, CC350, and CC1660) isolated in geographically distinct locations (Table S1). The majority of these strains were cultured from equid hosts, but the gene was also present in a few ruminant isolates. All isolates that harbored *scn-eq* also encoded *lukQ* and *lukP*, indicating that these virulence genes are prone to occur together. Even in isolates that do not encode a complete φSaeq1 prophage, as observed in two isolates from Brazilian buffalo (14), *scn-eq*, *lukQ*, and *lukP* are found in close proximity to each other on the chromosome. The sequence identity of *scn-eq* was highly conserved (>95%), and only a few SNPs were observed, which were associated with clonal lineage (Table S1). The *scn-eq* sequence of the CC1660 isolate showed the largest degree of variability and contained an insertion of nine base pairs, adding three amino acids (VKA) to the N terminus of eqSCIN. Altogether, we here describe a novel phage-encoded variant of SCIN associated with equid *S. aureus* isolates. The *scn-eq* gene co-localizes with the *lukPQ* genes and was found in all isolates encoding this equine-specific leukocidin.

### eqSCIN blocks complement activation in equine serum

**Figure 2.**
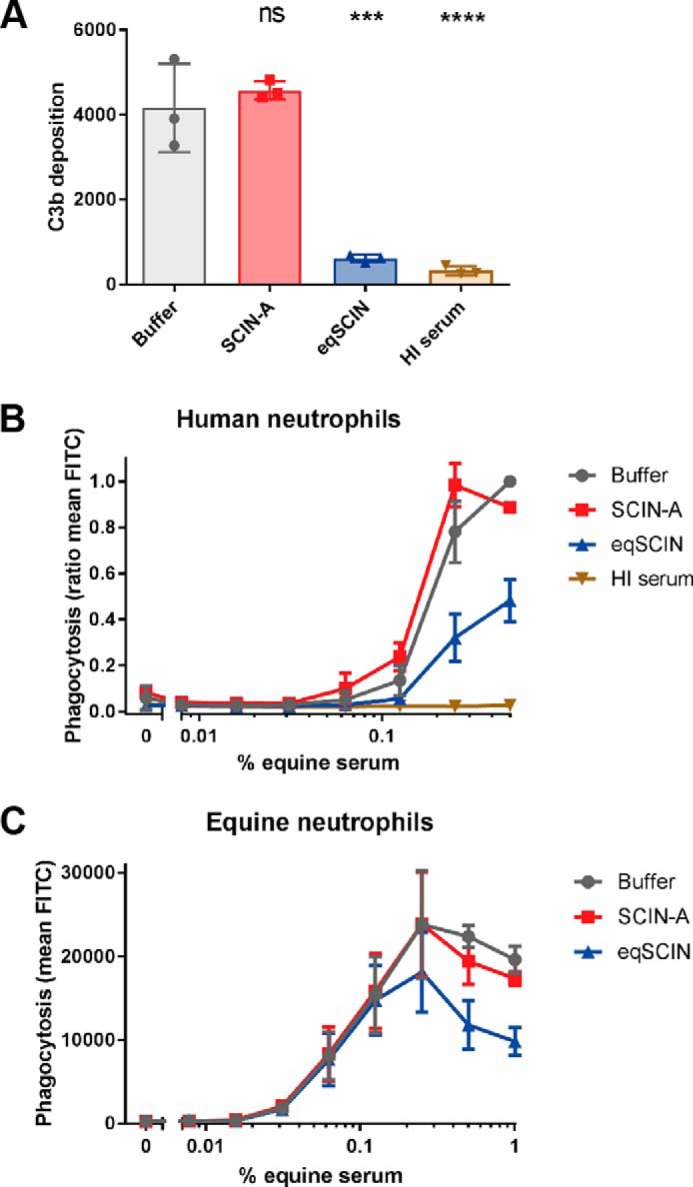
**eqSCIN blocks complement activation in equine serum.** *A*, eqSCIN inhibits C3b deposition on *S. aureus* in equine serum. Twenty percent horse serum was incubated with 30 nm SCIN-A or 37 nm eqSCIN, after which C3b deposition on cells of *S. aureus* strain Wood was measured via staining with anti-human C3b antibodies and flow cytometry. The extent of C3b deposition is shown as the geometric mean fluorescence of all gated bacteria. *B* and *C*, eqSCIN inhibits phagocytosis of *S. aureus* strain Wood by human (*B*) and equine (*C*) neutrophils after opsonization with a concentration range of horse complement. Preincubation of serum with SCIN-A shows no effect on phagocytosis. The amount of phagocytosis is shown as the ratio of mean FITC with 0.5% serum with buffer control set as 1 for *B* and mean FITC for *C. Error bars*, S.D.; *n* = 3. Significance relative to buffer control was determined by one-way ANOVA with Bonferroni post-test correction for multiple comparison. *ns*, non-significant; ***, *p* < 0.001; ****, *p* ≤ 0.0001.

To test whether eqSCIN indeed functions as a complement inhibitor, we first cloned and expressed the *scn-eq* gene (without the signal sequence) in *Escherichia coli* and purified the recombinant protein using nickel-affinity chromatography. Then we analyzed whether eqSCIN could block complement activity in equine serum. Because reagents for functional complement analyses in equine sera are limited, we developed a C3b opsonization assay on *S. aureus* in equine serum using flow cytometry. *S. aureus* (commonly used laboratory strain Wood) was incubated with equine serum, and deposition of C3b molecules onto staphylococci was determined via staining with anti-human C3b antibodies and flow cytometry. Representative flow plots of the assay are shown in Fig. S1. Western blotting was used to verify that polyclonal anti-human C3b antibodies cross-react with horse C3 (data not shown). In 20% equine serum, the antibodies detected C3b molecules on the bacterial surface, which was specific, because no signal was observed in serum that was heat-treated at 56 °C (to abrogate complement activity (15)). SCIN-A from human isolates effectively blocks C3b labeling in human serum (9, 16), but not in equine serum (Fig. 2*A*). In contrast, eqSCIN strongly inhibited C3b labeling by equine serum.

We next studied whether eqSCIN could affect the phagocytosis of *S. aureus* by neutrophils, which are critical immune cells in the first line of defense against *S. aureus* (17). To this end, GFP-labeled *S. aureus* strain Wood was preincubated with equine serum as a complement source, after which freshly isolated neutrophils were added for 15 min. We used human and equine neutrophils for our phagocytosis assays and showed that equine complement was competent for promoting phagocytosis by both human and equine neutrophils (Fig. 2, *B* and *C*). Again, we used heat-inactivated (HI) serum as a control to show that bacterial phagocytosis was indeed complement-dependent. Previous studies have established that SCIN-A from human isolates potently inhibits phagocytosis in the presence of human serum (9). In concordance with the C3b deposition experiments, we likewise observed that this SCIN-A does not interfere with phagocytosis in the presence of equine serum. However, the eqSCIN variant inhibited phagocytosis in equine serum (Fig. 2, *B* and *C*). Altogether, these data show that eqSCIN, in contrast to human SCIN-A, blocks complement activity in equine serum.

### eqSCIN interferes with equine C3 convertases

Previous investigations from our groups showed that SCIN-A blocks C3b deposition via a specific interaction with the C3 convertase enzymes (11). In the alternative pathway (AP), the C3 convertase consists of a transiently stable complex between surface-attached C3b and protease Bb (C3bBb) (18). For C3 cleavage to occur in this context, C3bBb first forms a 1:1 complex with its C3 substrate, and then Bb reorients itself into proximity with the scissile bond of C3 (by virtue of Bb binding a flexible domain of C3b). From structural studies of SCIN-A in complex with C3bBb (11), C3b, and its C3c fragment (19), we now know that the SCIN-A molecule makes ∼1,400 Å^2^ of contact with both C3b and protease Bb. This bipartite interaction blocks movement of Bb toward its substrate, thus rendering the enzymatic complex inactive.

**Figure 3.**
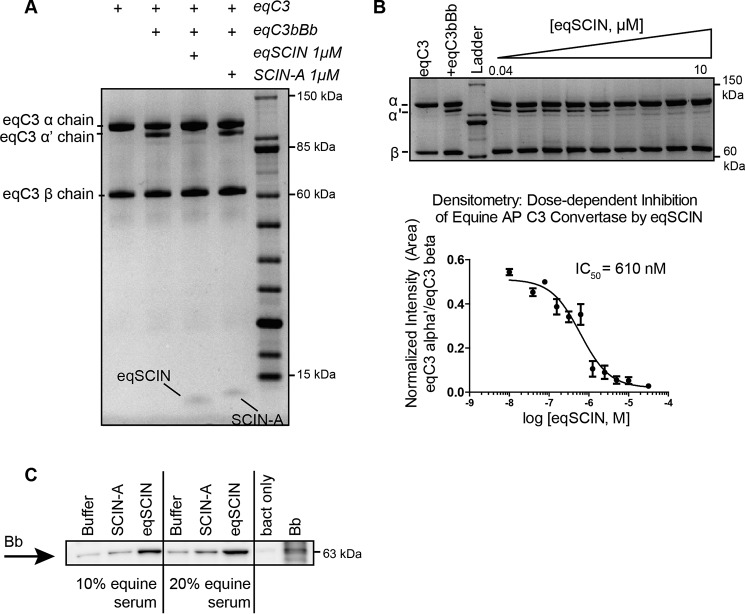
**eqSCIN interferes with equine C3 convertases.** *A*, the ability of eqSCIN or SCIN-A to inhibit the fluid-phase equine AP convertase was assessed. An equimolar solution of eqC3b and equine factor B was mixed with human factor D to form an equine AP convertase (eqC3bBb). Equine C3 was mixed in the presence or absence of 1 μm eqSCIN or SCIN-A for 1 h at 37 °C_._ The conversion of eqC3 α-chain to α′ was monitored on a reducing SDS-polyacrylamide gel. In the presence of 1 μm eqSCIN, this conversion is inhibited, whereas in the presence of SCIN-A, the reaction proceeds in a manner similar to buffer control. *B*, a 2-fold serial dilution of eqSCIN (0.04–10 μm) was incubated with eqC3bBb, and the band corresponding to the eqC3 α′-chain was quantified by densitometry using ImageJ, where the invariant eqC3 β-chain was used to normalize each lane. These data indicate that whereas eqSCIN inhibits equine AP convertases, SCIN-A does not. All assays were performed in duplicate, and the half-maximal inhibitory concentration (IC_50_) was calculated using variable non-linear regression in GraphPad Prism version 5. *C*, eqSCIN stabilizes Bb on the surface of bacteria opsonized with 10 and 20% equine serum, and SCIN-A does not. Buffer only and 1 μm SCIN-A showed background levels of Bb, whereas Bb stabilization was increased by adding 1 μm eqSCIN. One representative experiment is shown of three independent experiments.

To study whether eqSCIN blocks the equine AP C3 convertase in a similar manner, we first purified C3, C3b, and factor B from equine plasma using a modified purification protocol for human complement components. We then developed an assay in which activity of equine AP C3 convertase could be measured using these purified components. In this system, C3 conversion was assessed by adding equine C3 (eqC3) as a substrate to equine AP C3 convertase (eqC3bBb) that had been produced by mixing a solution of equine C3b (eqC3b), equine factor B, and human factor D. Inhibition of equine C3 cleavage was seen with eqSCIN, whereas SCIN-A failed to block eqC3 conversion (Fig. 3*A*). We further characterized the dose-dependent inhibition of eqC3bBb across eqSCIN concentrations ranging from 0.04 to 10 μm, which yielded a half-maximal inhibitory concentration (IC_50_) of 0.61 μm (Fig. 3*B*). These data strongly suggest that eqSCIN binds and interferes with activation of eqC3bBb.

Because C3bBb has a short half-life that causes rapid dissociation of Bb, this protease is not normally detected on target surfaces following opsonization. The binding of SCIN to C3bBb was found to stabilize this otherwise labile complex (11). We therefore examined whether eqSCIN, like human SCIN-A, could also stabilize C3bBb enzymes on bacterial surfaces. To address this question, we incubated *S. aureus* cells with equine serum in the presence of eqSCIN, and, after washing, surface-bound Bb molecules were detected using Western blotting. Whereas human SCIN-A did not affect eqC3bBb stability at the bacterial surface, we found that eqSCIN led to stabilization of convertases on *S. aureus* cells (Fig. 3*C*). Together, these data indicate that eqSCIN blocks complement activation in equine serum by stabilization of an inhibited form of the equine AP C3 convertases.

### eqSCIN binds equine and human C3b

**Figure 4.**
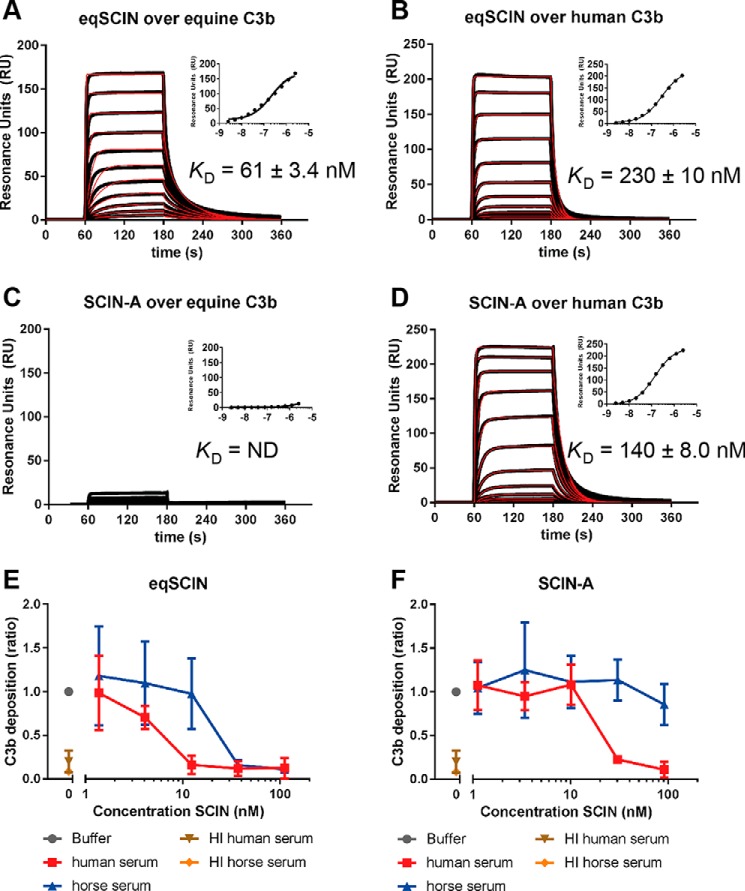
**eqSCIN binds equine and human C3b.** *A–D*, characterization of eqSCIN (*A* and *B*) or SCIN-A (*C* and *D*) binding to human C3b or eqC3b by SPR. eqSCIN binds to both equine and human C3b, whereas SCIN-A binds to only human C3b. *E*, eqSCIN inhibits C3b deposition on *S. aureus* in both 20% human and 20% horse serum, whereas SCIN-A (*F*) inhibits C3b deposition only in human serum. As control, HI serum from both human and horses does not lead to C3b deposition.

Following verification that eqSCIN indeed functions as a convertase inhibitor in horses, we wondered whether this molecule has similar host-restricted specificity as human SCIN-A molecules. We therefore performed surface plasmon resonance (SPR) to compare the affinity of eqSCIN with C3b molecules from equine and human origin (Fig. 4 (*A* and *B*) and Table 1). C3b was non-covalently captured on streptavidin biosensor chips using a previously described method where biotin is site-specifically linked to the thioester domain of C3b that normally anchors C3b to a target surface (12, 13). We found that eqSCIN binds with a comparably high affinity to both eqC3b and human C3b (*K_D_* = 61 and 230 nm, respectively) (Fig. 4, *A* and *B*). In concordance with previous reports, we observed that SCIN-A binds very weakly to eqC3b (*K_D_* = ND) but quite highly to human C3b (*K_D_* = 140 nm) (Fig. 4 (*C* and *D*) and Table 1).

**Table 1:** **Surface plasmon resonance: SCIN/C3b binding parameters** *K_D_*^SPR,kin^, equilibrium dissociation constant derived from rate constants (*k_d_*/*k_a_*); *K_D_*^SPR,ss^, equilibrium dissociation constant derived from steady-state fitting; ND, not determined; could not be calculated.

Immobilized ligand	Analyte	*K*D^SPR,kin^	*k_a_*	*k_d_*	*K*D^SPR,ss^
		*nm*	*m*^−*1*^ *s*^−*1*^	*s*^−*1*^	*nm*
Equine C3b	eqSCIN	61 ± 3.4	(1.8 ± 1.0) × 10^6^	(1.1 ± 0.65) × 10^−1^	220 ± 6.0
	SCIN-A	ND	ND	ND	29,000 ± 1700
Human C3b	eqSCIN	230 ± 10	(5.5 ± 0.01) × 10^5^	(1.3 ± 0.04) × 10^−1^	300 ± 12
	SCIN-A	140 ± 8.0	(6.3 ± 2.6) × 10^5^	(9.2 ± 4.3) × 10^−2^	140 ± 4.0

These C3b-binding analyses suggest that SCIN-A is specific for human complement and that eqSCIN might have activity against both human and equine complement. To investigate this further, we examined the ability of eqSCIN to inhibit the human complement system. We first measured C3b deposition on *S. aureus* cells using human and horse sera in the presence or absence of eqSCIN and SCIN-A (Fig. 4, *E* and *F*). In agreement with the SPR results, 100 nm eqSCIN fully inhibited (AP-dependent) C3b deposition in human and horse serum (Fig. 4*E*), whereas an identical concentration of SCIN-A only blocked C3b deposition by human serum (Fig. 4*F*). Examination of eqSCIN and SCIN-A across a range of concentrations revealed that eqSCIN (IC_50_ = 5.1 nm) (Fig. 4*E*) was 5 times more potent as an inhibitor of human complement compared with SCIN-A (IC_50_ = 26.1 nm) (Fig. 4*F*).

### eqSCIN blocks human, equine, and pig complement

**Figure 5.**
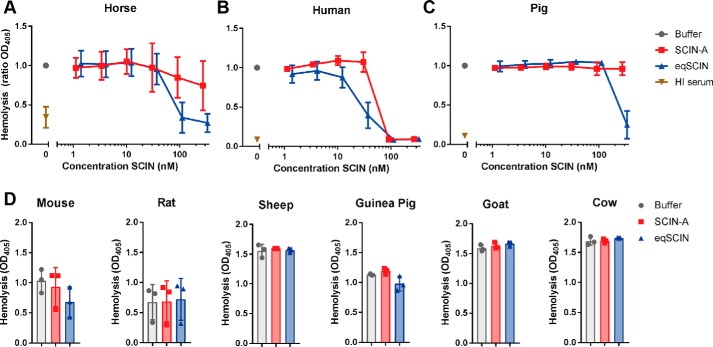
**eqSCIN blocks hemolysis in horses, humans, and pigs.** eqSCIN is able to inhibit AP-dependent hemolysis of erythrocytes caused by the complement system of pigs (*A*), horses (*B*), and humans (*C*), whereas SCIN-A inhibits the complement system of only humans (*C*). As a control, HI serum did not lead to hemolysis of the erythrocytes. *D*, both SCIN-A and eqSCIN (concentration of 1 μm was used) do not significantly block hemolysis in mouse, rat, sheep, guinea pig, goat, and cow serum. *Error bars*, S.D.; *n* = 3. Significance relative to buffer control was determined by one-way ANOVA with Bonferroni post-test correction for multiple comparisons.

The C3b-binding data described above suggested that eqSCIN is capable of inhibition of the complement system of multiple host species. To further explore the species specificity of eqSCIN, we used an assay of complement-dependent erythrocyte lysis to also test sera of other animals (mouse, rat, sheep, guinea pig, goat, cow, and pig). We initially verified that eqSCIN could inhibit complement activation in the presence of both equine (Fig. 5*A*) and human serum (Fig. 5*B*) in this assay, whereas SCIN-A only blocked human complement effectively. We also found that eqSCIN inhibited complement activity in pig serum (Fig. 5*C*), albeit at only the highest concentration of inhibitor tested. Testing a broader range of animals (mouse, rat, sheep, guinea pig, goat, and cow) revealed that eqSCIN only blocked equine, human, and pig complement, whereas SCIN-A inhibited the human complement system exclusively (Fig. 5*D*). As a control, HI serum did not show any hemolysis (Fig. S2). Together, these data established that eqSCIN is not specific for equine complement but also targets the AP C3 convertase of humans and pigs as well.

## Discussion

In this study, we identified a variant of the *S. aureus* virulence factor SCIN-A in equid isolates. Interestingly, we observed that eqSCIN was 5 times more potent as an inhibitor of human complement compared with SCIN-A (Fig. 4, *E* and *F*). This could be explained by the fact that SCIN has two major interaction sites in the C3 convertase (C3bBb). In contrast to SCIN-A orthologues found in human *S. aureus* isolates that only block human complement (16), this newly identified eqSCIN acts on a broader range of host species and is able to inhibit equine, human, and pig complement. These data on eqSCIN support previously observed broad species specificities of equine-adapted *S. aureus* immune evasion proteins (14, 20). Previous work on staphylococcal leukocidins and clotting factors in equid *S. aureus* isolates has identified that equine-adapted immune evasion proteins are not strictly horse-specific but have the ability to interact with immune mediators of multiple host species. For example, the equine-adapted leukocidin LukPQ kills equine neutrophils with higher efficiency than its closest relative in human isolates (LukED) (14). However, LukPQ also has the ability to lyse neutrophils of other animals (humans and cows), albeit less efficiently, in contrast to the φSa2-encoded leukocidin PVL that specifically kills human neutrophils (21). Similarly, a broader host range was observed for the equine variant of the von Willebrand factor–binding protein compared with similar molecules from ruminant strains (20). The observed broader species specificity of equine virulence factors like eqSCIN as opposed to the human-specific activity of molecules like SCIN-A is interesting and may result from similarities between the complement proteins of these hosts or possibly result from more frequent/recent host equine/human host jumps of these *S. aureus* lineages. Further research may be able to address these questions. Genome sequencing of wider collections of *S. aureus* strains isolated from equids over time will help to further investigate the evolution of equid-associated *S. aureus* lineages and their virulence factors.

Whereas SCIN-A and eqSCIN are only 58% identical (Fig. 1*B*), human C3b and eqC3b share nearly 80% identity at the amino acid sequence level. We were interested in understanding whether differences in amino acids on either side of the SCIN-A/C3b interface may provide an underlying structural explanation for the human-specific activity of SCIN-A. Previous work in our laboratories has produced crystal structures for SCIN-A, SCIN-A–C3bBb, SCIN-A–C3b, and SCIN-A–C3c (11, 22, 23). These studies have been extended to naturally occurring SCIN variants found in human-associated staphylococcal isolates and include atomic-resolution structures of an inactive SCIN protein known as SCIN-D (12, 24). We leveraged this information to model a putative SCIN-A–eqC3b complex. In the context of our previous structural and biochemical studies on SCIN-A–human C3b this approach afforded several potential insights into the human-specific nature of SCIN-A.

**Figure 6.**
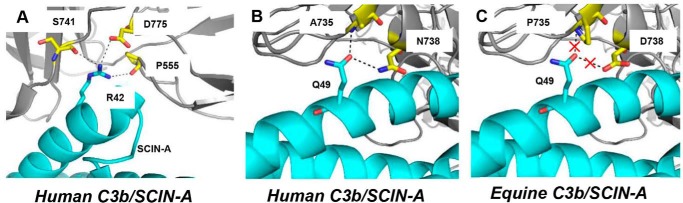
**Modeling a putative SCIN-A/eqC3b interface.** To gain insight into the human-specific nature of SCIN-A activity, the co-crystal structures of SCIN-A (*cyan*) in complex with human C3b (*gray*) were used to model an interaction between equine C3b and SCIN-A. *A*, SCIN-A Arg-42 forms hydrogen bonds with three human C3b residues (Pro-555, Ser-741, and Asp-775, marked in *yellow*) and is critical for mediating high-affinity SCIN-A/C3b interaction. An arginine residue is encoded at an equivalent position in eqSCIN, and C3b residues that directly contact Arg-42 are conserved between equine and human C3b. *B*, in contrast to the conserved Arg-42–mediated interaction, the Gln-49 residue of SCIN-A forms a salt bridge with human C3b residues Ala-735 and Asn-738 (shown in *yellow*), whereas in equine C3b (*C*), this interaction would be abrogated by A735P and N738D substitutions. The equivalent eqSCIN position encodes a Tyr residue rather than a Gln.

First, we noted that a key SCIN-A/C3b interaction involving SCIN-A Arg-42 (25) is conserved in the eqSCIN–eqC3b model (Fig. 6*A*). Despite its relative importance in driving overall C3b affinity, multiple lines of evidence from our studies involving SCIN variants indicate that residues other than Arg-42 are also critical in mediating C3b interaction and complement inhibitory activity (25). This point is underscored by the observation that SCIN-D, which possesses an equivalently positioned Arg residue, fails to interact with C3b and does not inhibit complement (11, 12). Next, we examined the C3b side of the interface. Of the 12 residues that bury more than 10% of total surface area in the SCIN-A–C3c crystal structure (as judged by the EBI PISA server (26) using Protein Data Bank entry 3L3O), four are non-conserved in eqC3b. These include human to horse substitutions of V554L, A735P, N738D, and V740I. Whereas Val-554 contacts SCIN-A Tyr-39, Val-740 makes contact with side-chain atoms of SCIN-A Lys-41 and Arg-42. Each of these SCIN-A residues is conserved in eqSCIN, and analysis of a putative SCIN-A/eqC3b interface reveals that the similar hydrophobic contacts mediated by each C3b residue are likely to be formed even with the V554L and V740I equine substitutions. Together, these observations suggest that these positions are unlikely to significantly alter the affinity of SCIN-A for eqC3b. In contrast, residues Ala-735 and Asn-738 mediate a salt-bridge interaction with SCIN-A Gln-49 (Fig. 6*B*). This interaction is abrogated in the SCIN-A–eqC3b model, as Pro-735 lacks the backbone amide and Asp-738 lacks the side-chain amino group that participate in the Gln-49 salt bridge (Fig. 6*C*). Interestingly, eqSCIN encodes a Tyr residue at this position and thus may be able to maintain high affinity for eqC3b via compensatory interactions.

It seems likely that subtle changes in structure resulting from altered sequences of SCIN proteins along with corresponding sequence differences in C3b give rise to their differing specificity profiles. Although our model seemingly implicates the A735P/N738D substitutions and the corresponding SCIN-A to eqSCIN Q49Y substitution, this alone does not explain the large differences in affinity that we observe for SCIN-A–C3b *versus* SCIN-A–eqC3b (data not shown). It is important to note several limitations inherent in this analysis. First, whereas we have only considered the primary SCIN-A–C3b site, a second SCIN-A–C3b–binding site involving residues of the N terminus of SCIN-A is present in all SCIN-A–C3b or C3c crystal structures. We have shown previously that this site contributes to the overall affinity of SCIN proteins for C3b (24), and candidate interactions analogous to the one described above can be identified (*i.e.* where substitutions on both sides of the SCIN/C3b interface are present). Second, an assumption of this type of analysis is that larger-scale structural changes are absent in eqSCIN or eqC3b relative to SCIN-A or human C3b, respectively. To this point, the crystal structure of the inactive SCIN-D protein revealed a change in the secondary structure of the second α-helix relative to SCIN-A as well as a significantly different surface charge profile (12). We cannot rule out the possibility that higher-order structural differences contribute to the C3b specificity observed for SCIN-A *versus* eqSCIN. Thus, future structure/function studies involving eqSCIN and eqC3b are needed to address the underlying structural basis for the human-specific activity of SCIN-A more comprehensively.

When considered as a whole, our functional experiments suggest an important role for eqSCIN in the evasion of the innate immune defense against *S. aureus* in horses. The close association of eqSCIN with LukPQ on phage φSaeq1 implies that these molecules may act in concert to evade the equine neutrophil response and contribute to *S. aureus* infections in horses. However, further evaluation of the clinical impact of both LukPQ and eqSCIN is clearly required to test this idea. Importantly, zoonotic transmission of equine isolates has been documented between hospitalized horses and their personnel (27–29). In Europe, an epidemic subclone of CC398 MRSA of almost exclusively spa-type t011 was shown to spread within and between equine hospitals (29). Interestingly, we demonstrated in our previous work that the prevalence of LukPQ in equid isolates of this spa type (t011) was relatively high (14). We can speculate that molecules like LukPQ and eqSCIN that block innate immune responses in horses and humans may facilitate zoonotic transmission of *S. aureus* in settings like the veterinary hospital. The same molecules may also facilitate host jumps between horses and other susceptible host species, like pigs (30). A follow-up study focusing on the population distribution of prophage φSaeq1 in *S. aureus* isolates of horses, humans, and pigs may reveal whether there is an association between φSaeq1 and *S. aureus* host switching, as was previously shown for other staphylococcal mobile genetic elements (31, 32). Although our understanding of *S. aureus* host adaptation is still largely incomplete, this study provides new insights into the ability of *S. aureus* to evade immune responses in a host-adaptive manner.

## Experimental procedures

### Bacterial strains and genomic analysis

The collection of sequenced *S. aureus* genomes used in this study has been described earlier in our previous work (14). In short, strains used in this study were collected as part of routine surveillance in the United Kingdom or were part of previous/ongoing studies in Switzerland (33), Tunisia (34, 35), and Brazil (36). BLASTn analysis was performed to identify the *scn-eq* gene in the genome collection, and the *scn-eq* sequence of isolate 3711 was used as a reference throughout the study. The complete sequence of the φSaeq1 phage of isolate 3711 is available in the Sequence Read Archive database of the European Nucleotide Archive (accession number LT671578). We furthermore used Wood 46 (ATCC-10832) for phagocytosis, C3b deposition, and convertase stabilization.

### Proteins

Recombinant SCIN with and without a His tag was prepared as described before (9). The protein without a His tag was used in the phagocytosis assay, whereas the His-tagged protein was used for the C3b deposition and hemolysis assay. A gel with the purified proteins is shown in Fig. S3. The eqSCIN coding sequence excluding the signal peptide sequence was amplified from strain 3711 using Phusion polymerase (Thermo Scientific). PCR products were ligated into a slightly modified expression vector pRSETB (Invitrogen Life Technologies) with a non-cleavable N-terminal His_6_ tag. Plasmids were transformed in *E. coli* (Rosetta-gami(DE3)pLysS (Novagen, Merck Biosciences)), and protein expression was induced using 1 mm isopropyl β-d-1-isogalactopyranoside. Bacterial pellets were lysed using 200 μg/ml lysozyme and three freeze-thaw cycles in 20 mm sodium phosphate (pH 7.8) to isolate the eqSCIN protein. His-tagged protein was purified using nickel-affinity chromatography (HiTrap chelating, HP, GE Healthcare) with an imidazole gradient ranging from 10 to 250 mm (Sigma-Aldrich). Finally, purified eqSCIN was dialyzed to PBS and stored at −20 °C.

### Phagocytosis and C3b deposition on S. aureus

Informed consent for blood draw was obtained from all subjects, in accordance with the Declaration of Helsinki. Approval from the medical ethics committee of the University Medical Center Utrecht was attained (METC-protocol 07-125/C approved on March 1, 2010). Blood was collected immediately upon death from four healthy horses during the slaughter process in tubes containing 3 mm EDTA anticoagulant. Equine neutrophils were isolated using 70% Percoll gradients as described before (37). Phagocytosis assays were carried out as described before (38). In short, 2.5 × 10^5^ cells of *S. aureus* Wood expressing GFP were incubated with 1 μm SCIN-A or eqSCIN (or buffer control without SCIN-A/eqSCIN), 0–0.5% (v/v) serum, and freshly isolated neutrophils for 15 min at 37 °C.

For C3b deposition, 20% horse and 20% human serum were preincubated with 30 nm SCIN-A or 37 nm eqSCIN and added to 2.5 × 10^7^ washed cells of *S. aureus* Wood WT. Surface-bound C3b was stained with anti-C3 conjugated with FITC (Protos Immunoresearch) and measured by flow cytometry analysis as geometric mean fluorescence of the gated bacteria. The buffer used for C3b deposition was veronal-buffered saline (140 mm NaCl), pH 7.4, 5 mm MgCl_2,_ 10 mm EGTA, and 0.05% (v/v) BSA.

### Conversion of C3

An equimolar solution of eqC3b and equine factor B (250 nm) was mixed with 100 nm human factor D to form an equine AP convertase (eqC3bBb). Equine C3 (2 μm) was mixed in the presence or absence of 1 μm eqSCIN or SCIN-A for 1 h at 37 °C in 20 mm HEPES (7.3), 140 mm NaCl, 5 mm NiCl_2_. The conversion of eqC3 α-chain to α′ was monitored on a reducing SDS-polyacrylamide gel. A 2-fold serial dilution of eqSCIN (0.04–10 μm) was incubated with eqC3bBb, and the band corresponding to the eqC3 α′-chain was quantitated by densitometry using ImageJ with the invariant eqC3 β-chain to normalize each lane. All assays were performed in duplicate, and an IC_50_ was calculated using variable non-linear regression in GraphPad Prism version 5.

### Convertase stabilization

Convertase C3bBb stabilization experiments were done as described before (9). In short, 2.5 × 10^7^ cells of *S. aureus* Wood were incubated for 20 min at 37 °C in veronal-buffered saline, pH 7.4, containing 1 mm MgCl_2_ and 1 mm CaCl_2_ plus 0.1% (v/v) BSA with different concentrations of horse serum and 1 μm SCIN-A, eqSCIN, or buffer control). After centrifugation, bacterially associated proteins were separated by SDS-PAGE, followed by immunoblot. Horse Bb was detected with goat anti-human factor B (Complement Technology) followed by peroxidase-conjugated anti-goat IgG (Santa Cruz Biotechnology, Inc.).

### Hemolysis assay

The alternative pathway hemolytic assay was performed by incubating 20% serum of different animals (30% for horse serum) with various concentrations of SCIN-A or eqSCIN (0–1 μm) or one fixed concentration of 1 μm and 2 × 10^7^ rabbit erythrocytes (Biotrading) for 1 h at 37 °C in veronal-buffered saline containing 5 mm MgCl_2_ and 10 mm EGTA. *A*_450_ nm was measured from supernatant of lysed cells.

### Protein-protein interaction assays by SPR

Direct binding of SCIN-A and eqSCIN to human or equine C3b was assessed by SPR on a Biacore T200 instrument. All experiments were performed at 25 °C in a running buffer of 20 mm HEPES (pH 7.3), 140 mm NaCl, 0.005% (v/v), Tween 20 (HBS-T) using a flow rate of 30 μl min^−1^. Site-specifically biotinylated human or equine C3b was prepared using protocols described previously (12, 13). C3b surfaces were prepared by capturing biotinylated C3b on a CMD-200 sensor chip (Xantec) that had previously been coupled with neutravidin (Sigma-Aldrich). eqC3b-biotin was captured at 3,000 resonance units and human C3b-biotin at 4,200 response units. A reference surface was generated by injecting with biotin only. SCIN-A or eqSCIN was injected for 2 min over each C3b surface in 2-fold serial dilutions ranging from 2.5 to 2,500 nm. The dissociation of SCIN–C3b complexes was monitored for 3 min. Baseline signal was achieved without the use of regeneration solutions. Each injection series was performed in triplicate. Data were fit to a 1:1 kinetic model of binding (*red lines*) as well a steady-state binding model (*inset plots*) using BiaEvaluation T200 software. In the case of SCIN-A–eqC3b, data could not be fit to a kinetic model, and thus a steady-state affinity was estimated. As the concentrations used for SCIN-A–eqC3b were subsaturating, this was achieved by fitting these data using a constant maximal observable signal (*R*_max_) based on the eqSCIN/eqC3b interaction.

### Statistical analyses

Statistical analysis was performed with GraphPad Prism version 7. Statistical significance was calculated using one-way ANOVA and Student's *t* test.

## Author contributions

N. W. d. J. and M. V. conceptualization; N. W. d. J., M. V., B. L. G., M. B., P. C. A., M. R., and B. V. G. data curation; N. W. d. J., M. V., B. L. G., G. K., J. A. v. S., M. H., E. M. H., B. V. G., and S. H. R. formal analysis; N. W. d. J., M. V., B. L. G., G. K., M. B., P. C. A., M. R., J. A. v. S., M. H., E. M. H., B. V. G., and S. H. R. investigation; N. W. d. J., M. V., B. L. G., M. B., P. C. A., M. R., and B. V. G. methodology; N. W. d. J., M. V., B. L. G., G. K., B. V. G., and S. H. R. writing-original draft; N. W. d. J. and S. H. R. project administration; B. L. G. software; G. K., J. A. v. S., M. H., E. M. H., B. V. G., and S. H. R. writing-review and editing; J. A. v. S., B. V. G., and S. H. R. supervision; E. M. H., B. V. G., and S. H. R. funding acquisition.

## Supplementary Material

Supporting Information

## Footnotes

4 The abbreviations used are:

SCIN: staphylococcal complement inhibitor
HI: heat-inactivated
AP: alternative pathway
SPR: surface plasmon resonance
ANOVA: analysis of variance.
