# Supplementary material for: Identification of a staphylococcal complement inhibitor with broad host specificity in equid Staphylococcus aureus strains
Source: J Biol Chem. 2018 Feb 5;293(12):4468–77. doi: 10.1074/jbc.RA117.000599 (PMC5868266; doi:10.1074/jbc.RA117.000599)
Supplement: Supporting Information [file supp_293_12_4468__index.html]

Identification of a Staphylococcal Complement Inhibitor with broad host specificity in equid S. aureus strains — Identification of staphylococcal equine SCIN — Identification of a staphylococcal complement inhibitor with broad host specificity in equid Staphylococcus aureus strains — Identification of staphylococcal equine SCIN — Supporting Information 

# Identification of a staphylococcal complement inhibitor with broad host specificity in equid *Staphylococcus aureus* strains

## Supporting Information

- Supporting information - This file contains one Supplemental Table, as well as three Supplemental Figures and their legends
