# Supplementary material for: Identification of a staphylococcal complement inhibitor with broad host specificity in equid Staphylococcus aureus strains
Source: J Biol Chem. 2018 Feb 5;293(12):4468–77. doi: 10.1074/jbc.RA117.000599 (PMC5868266; doi:10.1074/jbc.RA117.000599)
Supplement: Supporting Information [file supp_RA117.000599_133400_1_supp_61591_p32c8c.pdf]

Supporting information

Identification of a Staphylococcal Complement Inhibitor with broad host specificity in equid *S. aureus* strains

Nienke W.M. de Jong[1*], Manouk Vrieling[1,2*], Brandon L. Garcia[3], Gerrit Koop[4], Matt Brettmann[3], Piet C. Aerts[1], Maartje Ruyken[1], Jos A. G. van Strijp[1], Mark Holmes[5], Ewan M. Harrison[6], Brian V. Geisbrecht[3#] and Suzan H.M. Rooijakkers[1#,$]

From [1]Dept. of Medical Microbiology, University Medical Center Utrecht, 3584 CX Utrecht, The Netherlands;
[2]The Roslin Institute, University of Edinburgh, Easter Bush Campus, Midlothian EH25 9RG, UK;
[3]Dept. of Biochemistry & Molecular Biophysics, Kansas State University, Manhattan, KS 66506, USA;
[4]Dept. of Farm Animal Health, Faculty of Veterinary Medicine, Utrecht University, 3584 CL Utrecht, The Netherlands;
[5]Department of Veterinary Medicine, University of Cambridge, Cambridge CB3 0ES, UK;
[6]Department of Medicine, University of Cambridge, Addenbrooke's Hospital, Cambridge CB2 0QQ, UK

Running title: Identification of staphylococcal equine SCIN

[$] Author to whom correspondence should be addressed: Suzan H.M. Rooijakkers; E-mail s.h.m.rooijakkers@umcutrecht.nl; Department of Medical Microbiology, University Medical Center Utrecht and Utrecht University, Heidelberglaan 100, 3584 CX, Utrecht, the Netherlands Tel. +31 (0)887559962

Keywords: Complement system, Equine, Host adaptation, Immune evasion, SCIN

This file contains one Supplemental Table, as well as three Supplemental Figures and their legends

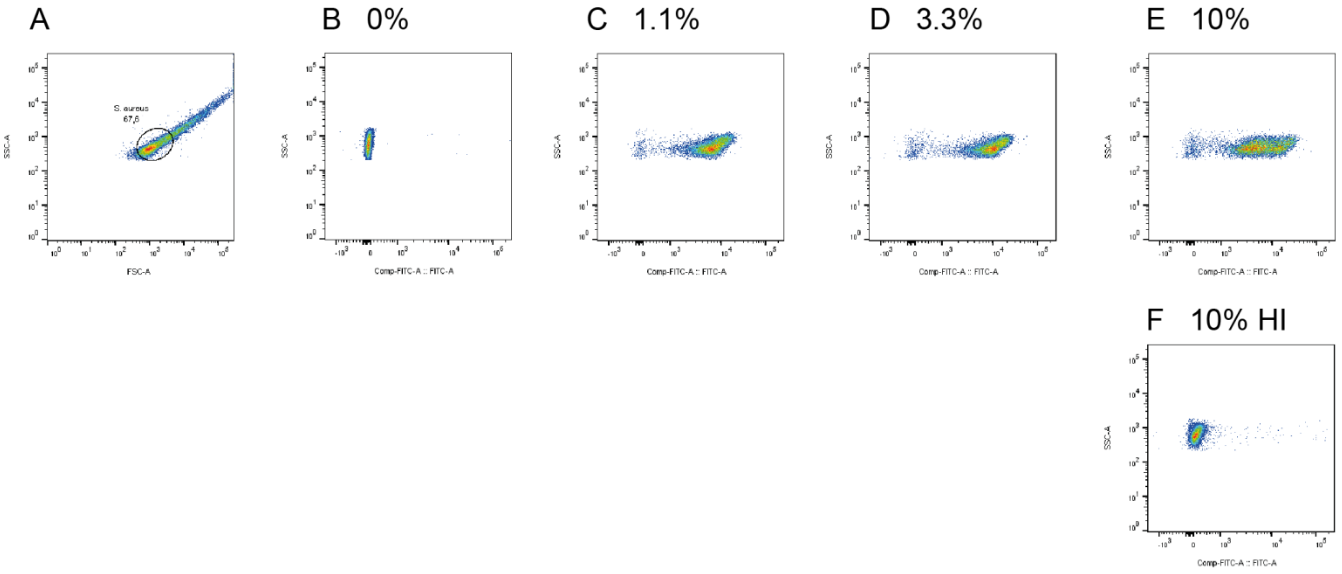

Figure S1. Representative flow plots for C3b deposition with equine serum. (A) Representation of gating *S. aureus* Wood in each well. (B-E) Detection of surface-bound C3b with anti-C3 conjugated with FITC and measured by flow cytometry analysis as geometric mean fluorescence of the gated bacteria after addition of various concentrations of equine serum. (F) 10% HI serum does show any C3b deposition, by the lack of FITC signal.

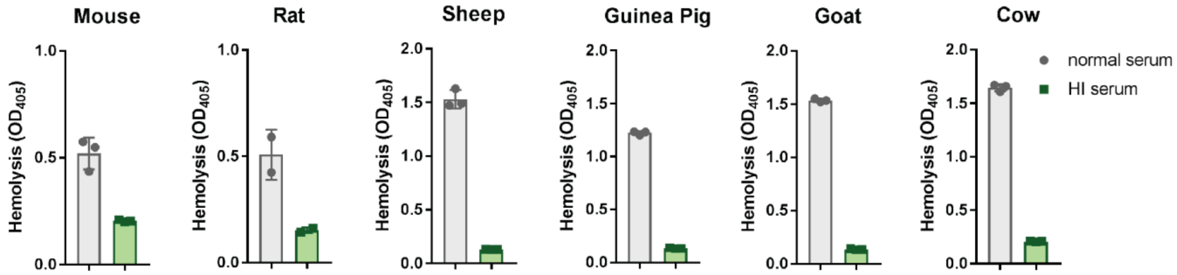

Figure S2. HI serum lacks ability to lyse erythroyctes compared to normal serum. The serum of the different animals used in Figure 5D were heat-inactivated and tested in the hemolysis assay. The complement in the sera of all animals lacked activity to lyse erythrocytes, while normal serum had active complement to lyse the erythrocytes.

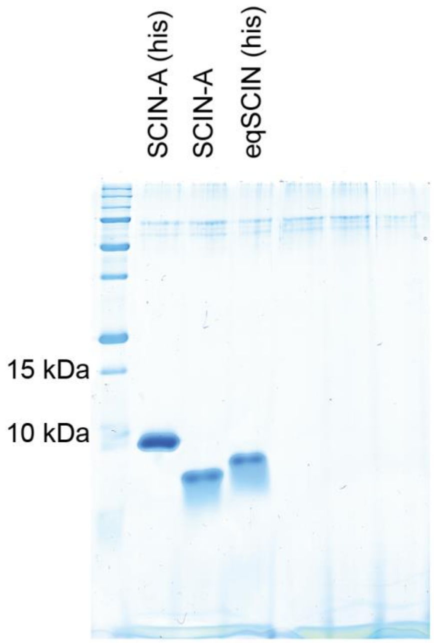

Figure S3. Protein gel with purified proteins used in this paper. Purified SCIN-A without his tag (lane 1) was used in the phagocytosis assay, while the his-tagged protein was used for the C3b deposition and hemolysis assay (lane 2). The protein in lane 3 is eqSCIN with non-cleavable 6xHis tag. The concentration of the proteins is 150 µg/ml.

Supplementary table 1: *scn-eq* is present in *Staphylococcus aureus* strains from different clonal complexes (CC) and different countries. For *scn-eq*, the gene location (prophage/chromosome), % identity to the reference (3711), single nucleotide polymorphisms (SNPs) and insertions (INS) are indicated. Furtermore, the presence of lukPQ and other scns are reported.

					scn-eq									*lukPQ*		
						SNPs							INS			
Strain ID	CC	Country	Host species	Location	Identity (%)	75^{ns}	103^{ns}	157^{ns}	189^{s}	198^{ns}	254^{ns}	318^{s}	87	*lukP*	*lukQ*	Reference
KM595-06	1	Switzerland	Horse	Prophage	98,261	G	A	G	A	C	G	T		+	+	Sieber *et al.* 2011 (34)
KM777-07	1	Switzerland	Horse	Prophage	98,261	G	A	G	A	C	G	T		+	+	Sieber *et al.* 2011 (34)
KM489-05	1	Switzerland	Horse	Prophage	98,261	G	A	G	A	C	G	T		+	+	Sieber *et al.* 2011 (34)
m-25-21 2.3	1	United Kingdom	Cow	Prophage	98,261	G	A	G	A	C	G	T		+	+	Koop *et al.* 2017 (14)
m-25-21 2.11	1	United Kingdom	Cow	Prophage	98,261	G	A	G	A	C	G	T		+	+	Koop *et al.* 2017 (14)
8205	1	United Kingdom	Horse	Prophage	98,261	G	A	G	A	C	G	T		+	+	Koop *et al.* 2017 (14)
1928	1	United Kingdom	Horse	Prophage	98,261	G	A	G	A	C	G	T		+	+	Koop *et al.* 2017 (14)
8231	1	United Kingdom	Horse	Prophage	98,261	G	A	G	A	C	G	T		+	+	Koop *et al.* 2017 (14)
8182	1	United Kingdom	Horse	Prophage	98,261	G	A	G	A	C	G	T		+	+	Koop *et al.* 2017 (14)
VetBz55B	133	Brazil	Buffalo	Chromosome	100	T	G	A	T	G	A	T		+	+	Aires-de-Sousa *et al.* 2007 (37)
VetBz63	133	Brazil	Buffalo	Chromosome	100	T	G	A	T	G	A	T		+	+	Aires-de-Sousa *et al.* 2007 (37)
c3388	133	Tunisia	Donkey	Prophage	100	T	G	A	T	G	A	T		+	+	Gharsa *et al.* 2012 (35)
c3401	133	Tunisia	Donkey	Prophage	100	T	G	A	T	G	A	T		+	+	Gharsa *et al.* 2012 (35)
c3403	133	Tunisia	Donkey	Prophage	100	T	G	A	T	G	A	T		+	+	Gharsa *et al.* 2012 (35)
c4439	133	Tunisia	Donkey	Prophage	100	T	G	A	T	G	A	T		+	+	Gharsa *et al.* 2012 (35)
c4444	133	Tunisia	Donkey	Prophage	100	T	G	A	T	G	A	T		+	+	Gharsa *et al.* 2012 (35)
c4445	133	Tunisia	Donkey	Prophage	100	T	G	A	T	G	A	T		+	+	Gharsa *et al.* 2012 (35)
c4451	133	Tunisia	Donkey	Prophage	100	T	G	A	T	G	A	T		+	+	Gharsa *et al.* 2012 (35)
c4452	133	Tunisia	Donkey	Prophage	100	T	G	A	T	G	A	T		+	+	Gharsa *et al.* 2012 (35)
c3815	133	Tunisia	Goat	Prophage	100	T	G	A	T	G	A	T		+	+	Gharsa *et al.* 2012 (35)
7107	133	United Kingdom	Horse	Prophage	100	T	G	A	T	G	A	T		+	+	Koop *et al.* 2017 (14)
7540	133	United Kingdom	Horse	Prophage	100	T	G	A	T	G	A	T		+	+	Koop *et al.* 2017 (14)
5431	133	United Kingdom	Horse	Prophage	100	T	G	A	T	G	A	T		+	+	Koop *et al.* 2017 (14)
8401	133	United Kingdom	Horse	Prophage	100	T	G	A	T	G	A	T		+	+	Koop *et al.* 2017 (14)
3711	133	United Kingdom	Horse	Prophage	100	T	G	A	T	G	A	T		+	+	Koop *et al.* 2017 (14)
8572	350	United Kingdom	Horse	Prophage	98,261	G	A	G	A	C	G	T		+	+	Koop *et al.* 2017 (14)
3507	522	United Kingdom	Horse	Prophage	99,42	T	G	A	A	G	A	C		+	+	Koop *et al.* 2017 (14)
8571	522	United Kingdom	Horse	Prophage	98,261	G	A	G	A	C	G	T		+	+	Koop *et al.* 2017 (14)
KM1549-2-06	1660	Switzerland	Horse	Prophage	95,763	G	A	G	A	C	G	T	AAAGC[illegible]	+	+	Sieber *et al.* 2011 (34)

^{ns} = non-synonymous

^{s} = synonymous
